# A first observation of spermatogenesis in mature male scalloped hammerheads (*Sphyrna lewini*) from Zinkwazi, KwaZulu-Natal, South Africa

**DOI:** 10.1007/s10695-020-00871-z

**Published:** 2020-09-11

**Authors:** Helené J. Coetzee, Kristina Naidoo, Ina Wagenaar

**Affiliations:** 1 Department of Zoology, University of Johannesburg, P.O Box 524, Auckland Park, Johannesburg, 2006 South Africa; 2 Research and Monitoring Division, KwaZulu-Natal Sharks Board, Private Bag 2, Umhlanga Rocks, Durban, 4320 South Africa

**Keywords:** Reproductive biology, Spermatogenesis, Histology, Diametrical testes, South Africa

## Abstract

Sharks are not only threatened, but also have a low fecundity as they are being overfished. The shark family, Sphyrnidae, consists of nine species of which three are found in South African oceans. One of the three Sphyrnidae species, the scalloped hammerhead (*Sphyrna lewini*) are the most common, but their biology and mode of reproduction are not extensively studied in terms of their reproductive biology. The aim of this study was to describe the germ cell development in the testes of sexually mature male scalloped hammerheads. Three individual male *S. lewini* were caught at Zinkwazi, KwaZulu-Natal, South Africa. The sharks and their reproductive organs were weighed and measured to collect the biometric data for the condition factor and the gonado-somatic index. Following standard necropsy, the testes were fixed in Bouin’s solution and processed for histological assessment. The histological assessment revealed that the testes of *S. lewini* consist of seminiferous tubules which form part of a larger lobular structure with germ cells in different stages of development, from spermatogonia to mature spermatozoa. Seven stages of development were identified during the process of spermatogenesis, similar to what has been described for elasmobranchs. In conclusion, this study provides evidence that the testes of *S. lewini* are diametrical and polyspermatocystic and conforms to the testes structure of elasmobranch males.

## Introduction

Sharks are a group of cartilaginous fish which originated around 400 million years ago and at present comprise of 500 extant species (Compagno et al. 2005). They range from apex predators (great white shark) to filter feeders (whale shark). The apex predators maintain the balance of the food web and serve as an indicator of ocean health (Griffen et al. 2008). Through spatial control and abundance, sharks have indirectly maintained seagrass beds, coral reefs and commercial fishing (Griffen et al. 2008). Sharks are threatened as they are being overfished and caught as by-catch which cause a decline in population numbers. In a recent study done by Cardeñosa (2019), various genetic material of threatened species of shark were found in pet food and cosmetic products. Fecundity amongst sharks are low which also plays a factor in their declining numbers (Hazin et al. 2001; De Bruyn et al. 2005).

There are 100 different shark species, widely distributed in Southern Africa, many species present in the sub-tropical waters of the Indian Ocean of which many are harmless (Compagno et al. 2005; Branch et al. 2017). The Sphyrnidae family consists of nine species of hammerheads of which three species namely the great hammerhead (*Sphyrna mokarran*), smooth hammerhead (*Sphyrna zygaena*) and *S. lewini* co-exist in South African waters (Compagno et al. 2005). *Sphyrna lewini* is commonly recorded from northern Mozambique to the south coast of KwaZulu-Natal (KZN), South Africa (Bass et al. 1975; De Bruyn et al. 2005) and is the third most common shark caught in the KZN bather protection gillnets overseen by KwaZulu-Natal Sharks Board (KZNSB) (De Bruyn et al. 2005).

Following a series of fatal bather shark attacks between 1940 and 1950, large bather protection gillnets were introduced to KZN, South Africa, in 1952 (Dudley and Simpfendorfer 2006). The continuation of a protection programme led to KwaZulu-Natal Sharks board being created in 1964 for the maintenance of these nets (Cliff and Dudley 1992; Dudley et al. 1998) in conjunction with attempts at reducing marine capture (Dudley and Cliff 1993; Dudley et al. 1998).

The International Union for Conservation of Nature (IUCN) has classified the *S. lewini* as endangered (Dulvy et al. 2014; ICUN 2014). It is also one of the most common sharks caught in the KZN bather protection nets (De Bruyn et al. 2005; Baum et al. 2009) and will therefore serve as the study species in this paper. These sharks are noticeably distinguished from the other elasmobranchs due to its iconic hammer-shaped head (McComb et al. 2009). Hammerheads are cephalofoils which gives them the ability of lateral projection with a 360° visual radius (McComb et al. 2009) enhancing their ability to capture prey and making them an important species within their ecosystems (McComb et al. 2009).

The *S. lewini* males are considered mature when they reach a total length (TL) of 140–165 cm (Compagno et al. 2005) and precaudal length (PCL) of 161 cm (De Bruyn et al. 2005). Reproduction in hammerheads are placental viviparous. Pratt (1988) described three types of testes in sharks based on the seminiferous follicle origin i.e. diametrical, compound and radial. In diametrical testes, the germinal zone is found disto-laterally and the seminiferous follicle development proceeds diametrically (meaning alongside the cross-sectional width of the testes towards the proximally located efferent ducts) (Pratt 1988) as opposed to compound testes when the germinal zone is found dorsally in the testes and as the spermatocytes develop, columns are formed in a radial pattern (Pratt 1988; Hamlett 2005). This type of seminiferous follicle origin development should not be confused with radial testes where the germinal zones of the testes are not found only in the dorsal side of the testes but throughout the testes (Pratt 1988; Hamlett 2005). As the spermatocytes developed, they move away from the germinal zone in a radial pattern (Pratt 1988; Hamlett 2005). Mating season for *S. lewini* occurs during spring to early/mid-summer on the South African coast (De Bruyn et al. 2005). If females are kept isolated from males, parthenogenesis can occur in *Sphyrna tiburo* (bonnet head shark) (Chapman et al. 2007).

The anatomical male reproductive biology of *S. lewini* has been described before (Pratt 1988; Girard et al. 2000; Jensen et al. 2002; Compagno et al. 2005; Conrath 2005; Hamlett 2005). The male reproductive systems compromise of diametrical testes (as previously described), epididymis, ductus efferens, seminal vesicles, ductus deferens, accessory glands and an internal pair of siphon sacs with a pair of claspers (urogenital papillae) found externally. Testes are the main reproductive organ and produce steroid hormones and androgens (Jensen et al. 2002; Conrath 2005; Hamlett 2005). A germinal layer is evident along the length of the testes which serves as the origin of spermatogenesis (Pratt 1988; Girard et al. 2000; Conrath 2005). Externally, male sharks have claspers (urogenital papillae) that calcify with maturity (Girard et al. 2000; Jensen et al. 2002) and act as a copulatory organ (Compagno et al. 2005). During copulation, the claspers of the male shark are placed inside the female’s cloaca to pass the semen via the groove on the clasper for internal fertilisation (Compagno et al. 2005; Conrath 2005; Hamlett 2005).

Spermatogenic development in all elasmobranches is considered polyspermatocystic meaning there are numerous amounts of seminiferous tubules inside each testes (Grier 1992). Spermatogenesis is the process of spermatozoa development from germ cells to mature spermatozoa in the seminiferous tubules within the testes. Shark testes consist of seminiferous tubules which form part of a larger lobular structure with germ cells in different stages of development (Pratt 1988; Grier 1992; Parsons and Grier 1992; Girard et al. 2000; McClusky and Sulikowski 2014; Do Rêgo et al. 2016). Germ cell development takes place in the seminiferous tubules, with initial development from the basal membrane on the periphery of the seminiferous tubule. The spermatogonia originate from the basal membrane of the seminiferous tubule through the process of mitosis (Parsons and Grier 1992; Girard et al. 2000; McClusky and Sulikowski 2014; Do Rêgo et al. 2016). Spermatogonia then undergo meiosis to form primary and secondary spermatocytes. Secondary spermatocytes undergo haploid division to form spermatids. Immature spermatozoa develop from the spermatids which then move to the periphery of the basal membrane (Parsons and Grier 1992; Girard et al. 2000; McClusky and Sulikowski 2014; Do Rêgo et al. 2016). Mature spermatozoa clump together to form spermatozeugmata which are distinctive spiral structures in the eosinophilic matrix to mature near the basal membrane of the seminiferous tubule (Parsons and Grier 1992; Girard et al. 2000; McClusky and Sulikowski 2014; Do Rêgo et al. 2016).

The general growth and health status of many fish species are determined using the condition factor (CF), which is calculated using the length/weight ratio of the individual (Carlander 1969; Adams et al. 1993; De Bruyn et al. 2005; Logan et al. 2018). The reproductive stages are determined using the gonado-somatic index (GSI) which is the relation of the gonad weight to the total body weight (Hazin et al. 2001; De Bruyn et al. 2005; Das Neves et al. 2018).

The testes histology and spermatogenesis of *S. lewini* from the south-west coast of Mexico has been described by Bejarano-Álvarez et al. (2010) but there exists a discrepancy in the present literature, terminology and histological testes structures. The spermatogenic stages of the closely related *S. tiburo* from the Gulf of Mexico were also described by Parsons and Grier (1992). Although Bejarano-Álvarez et al. (2010) referred to the stages of development, this study provides a more detailed account of the developing germ cells in the seminiferous tubules from *S. lewini*. The importance of the study is not only an expansion of existing literature and confirmation of the reproductive development data for *S. lewini,* but also contribute to the data for the family of Sphyrnidae. Thus the aim of the study is to describe the germ cell development and stages of development in the testes of mature male *S. lewini*.

This study provides the first detailed histological description of germ cell development in the testes of mature active *S. lewini* male shark in South Africa and contributes to the expansion and confirmation of mode of reproductive development.

## Material and methods

The study was ethically approved by the University of Johannesburg’s Ethical Committee (Ethics No: 23/05/2018).

### Study species

**Fig. 1:**
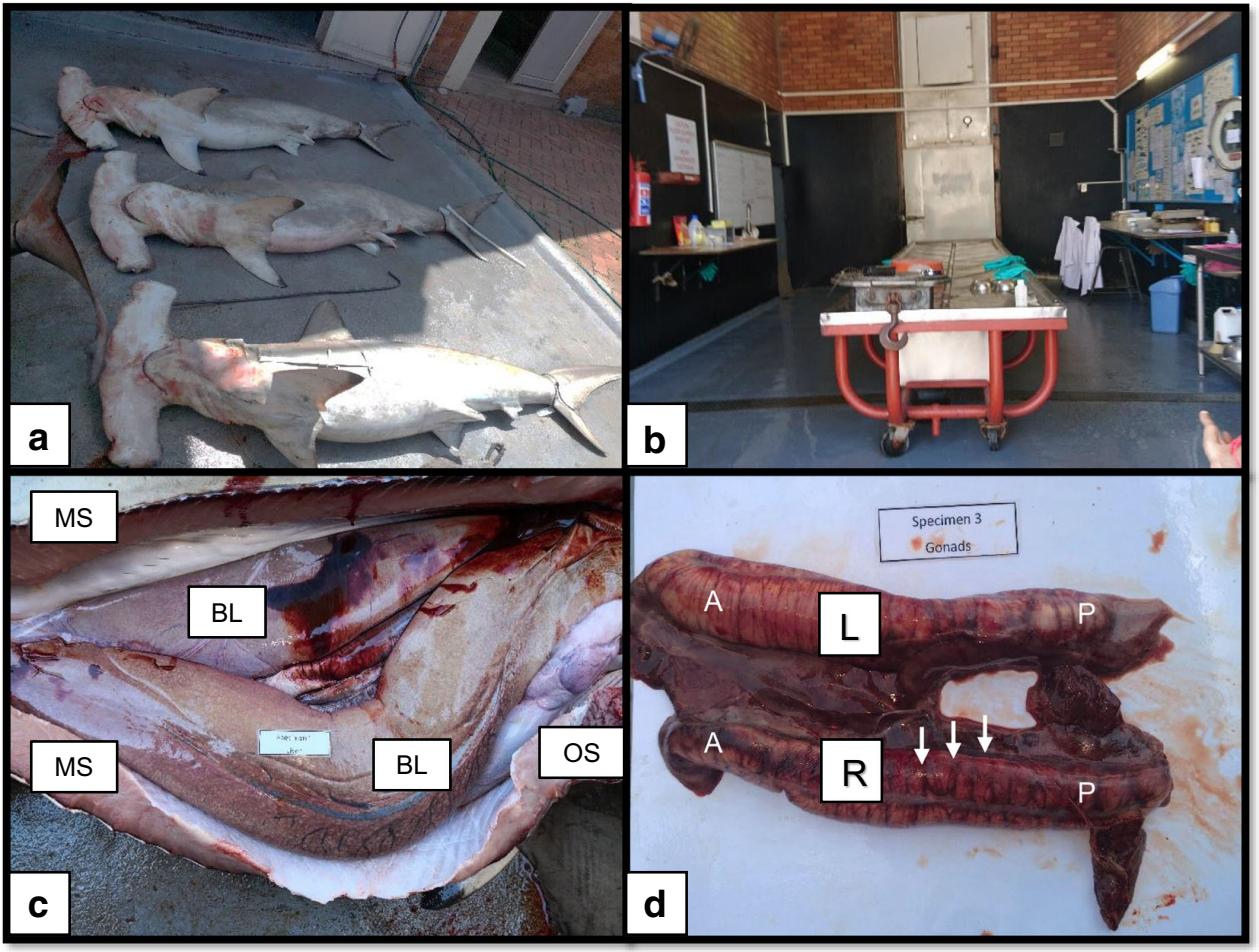
Images showing necropsy during field sampling: **a** Three mature male *S. lewini* captured at Zinkwazi, SA, **b** KZNSB Dissection Wet Laboratory, **c** Internal anatomy of a *S. lewini* during a standard necropsy showing the bi-lobed liver (BL) and exposed white muscle (MS) to the left and right sides of the incision point together with a partial view of the oesophagus (OS) (bring the OS block down as its obscuring the view of the oesophagus) and **d** A closer anterior (A) and posterior (P) view of the removed left (L) and right (R) testes compromising of lobules (which were the striations seen macroscopically indicated by the arrows)

Three mature and sexually active *S. lewini* males were caught by the KZNSB bather protection gillnets of the coast of Zinkwazi, KZN on the 27th of November 2018 (Fig. 1a). Specific details regarding the net installations, net services and operations are available (Cliff et al. 1990; Cliff and Dudley 1992; Dudley and Simpfendorfer 2006). The three specimens were transported to the KZNSB Wet Dissection Laboratory on the same day (in Umhlanga) (Fig. 1b). Specimens were initially evaluated for freshness, maturity and sexual classification based on classification system adapted from Stehmann (2002). Biometric data was collected from the *S. lewini s*pecimens by vertically weighing using a crane scale (DeMag C100), length of the body, testes and claspers were horizontally measured with a measuring tape (cm) while testes were weighed (g) using a balanced scale,

Using the biometric data, the condition factor (CF) was determined using the following formula by Carlander (1969):

$$ \mathrm{CF}=\frac{\mathrm{Weight}\ \left(\mathrm{g}\right)\ \mathrm{x}\ {10}^5}{\mathrm{Length}\ \mathrm{of}\ \mathrm{body}\ {\left(\mathrm{mm}\right)}^3} $$

The gonado-somatic index (GSI) was calculated using the following formula:

$$ \mathrm{GSI}=\frac{\left[\mathrm{Left}\ \mathrm{Testes}\ \mathrm{Weight}\ \left(\mathrm{g}\right)+\mathrm{Right}\ \mathrm{Testes}\ \mathrm{Weight}\ \left(\mathrm{g}\right)\ \mathrm{x}\ 100\right]}{\mathrm{Total}\ \mathrm{Weight}\ \left(\mathrm{g}\right)} $$

### Necropsy and sample collection

**Fig. 2:**
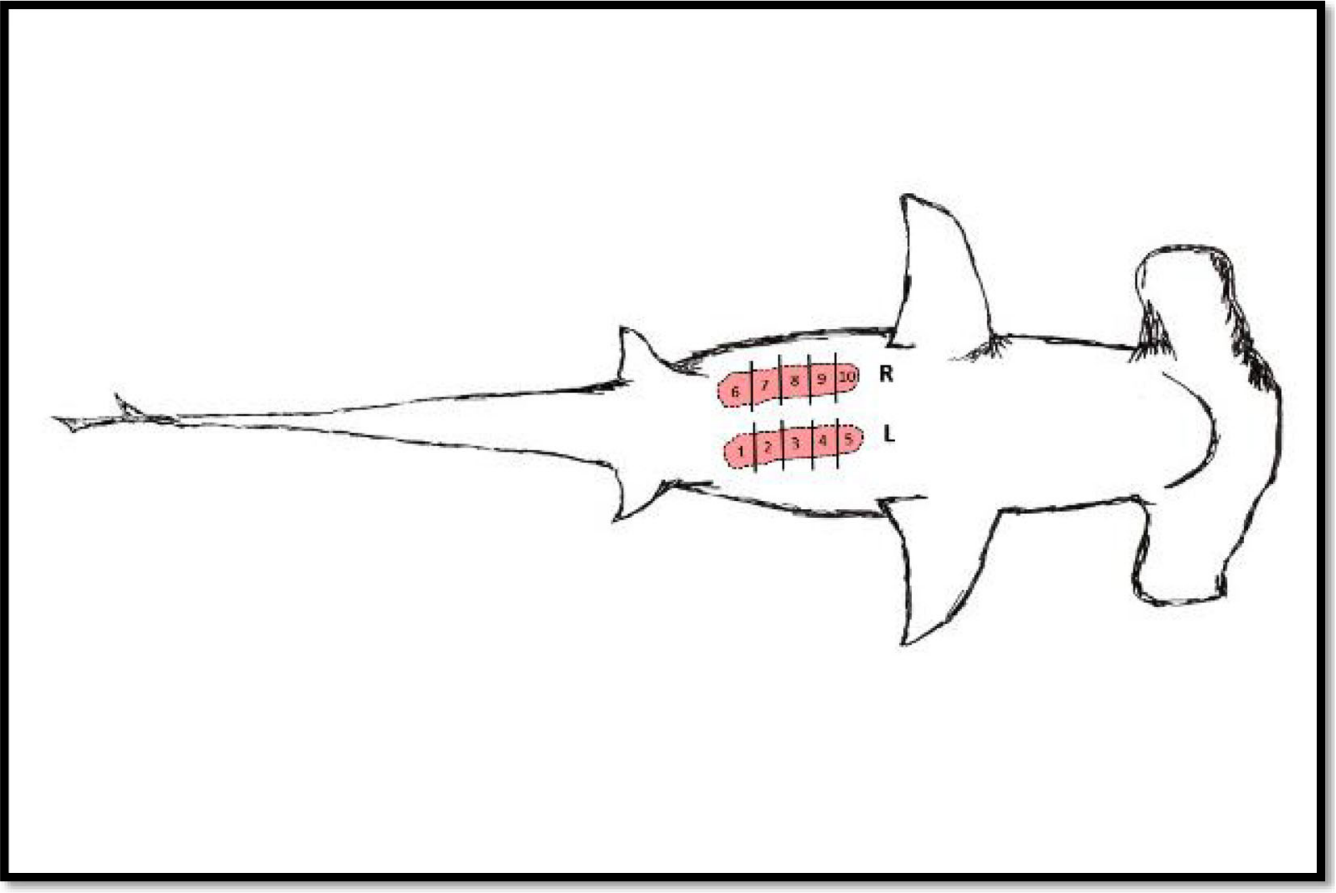
Illustration of *S. lewini* testes [Left (L) and Right (R)] to indicate the ten (1–10) sampling areas (personal illustration)

After collection of biometric data, a standard necropsy was performed on all three specimens to identify any internal and external macroscopic abnormalities (Fig. 1c). The left and right testes were removed from each specimen in order to be weighed and measured (Fig. 1d). Each pair of testes were divided into five equal sections respectively (Fig. 2). A 1 cm × 1 cm × 1 cm cube of testes tissue was sampled medially (to ensure that decayed tissue was not sampled) from both the right and the left testes sampling within the 5 different demarked areas (Fig. 2) on both the left and right pair of testes. Each area (left and right) was divided with a cross-section cut and collection of the 1 cm^3^ of tissue was removed from each centre of the represented area in a similar manner throughout. Testes tissues samples were collected anteriorly and posteriorly in order for the sample areas to be representative of the testes. A large number of samples were collected to ensure that the tissues are representative of the different areas of the testes, taking into consideration the large size of the testes and that different areas of the testes were found in different stages of spermatogenic development. Each testes tissue sample was fixed in Bouin’s solution for 24 h before washing the tissue in running tap water.

### Tissue processing, sectioning and staining

After fixation, the testes samples were dehydrated in ascending series of ethanol from 30 to 100%. Tissue samples were cleared in xylene and prepared for embedding in paraffin wax. Wax bocks were sectioned (5 μm) with a Reichert Jung microtome and mounted onto microscope slides. The slides were stained using haematoxylin and eosin (H&E) following standard protocols (Humason 1979; Van Dyk and Pieterse 2008; Das Neves et al. 2018) and coverslips were mounted.

### Histological light microscopy assessment

Histological assessment of the testes tissues was done using a Leica Light Microscope 020-518.500, a Leica DMC 2900 Camera and the Multi-Headed Olympus Camera Viewer Program. The scalebar for the micrographs in this study represented the actual size of the bar and was thus used to compare tissue and cell structure.

## Results

### Biometric data

Table 1 presents the summarised biometric data of the three sexually mature *S. lewini* (specimen 1–specimen 3).

**Table 1:** Biometric data for *S. lewini*; total weight (kg), total length (cm), clasper lengths (mm), clasper widths (mm), testes weight (g), testes length (mm), condition factor (CF) and the gonado-somatic index (GSI, %)

Shark	Total weight (kg)	Total length (cm)	Clasper length (mm)		Clasper width (mm)		Testes weight (g)		Testes length (mm)		CF	GSI (%)
			L	R	L	R	L	R	L	R		
Specimen 1	180	253.4	246	244	55	54	154.6	167.4	225	266	1.11	0.18
Specimen 2	110	217.0	228	231	65	73	205.4	176.5	283	319	1.10	0.35
Specimen 3	86	260.0	228	204	40	45	180	176	235	243	0.49	0.41
Mean	125.33	243.47	234.00	226.33	53.33	57.33	180.00	173.30	247.67	276.00	0.89	0.31
Standard deviation	48.84	23.16	10.39	20.40	12.58	14.29	25.40	5.12	31.01	38.97	0.35	0.12

CF condition factor, *GSI* gonado-somatic index measured in %, *L* left side, *R* right side measured in mm

All specimens were categorised as sexually mature and active based on overall assessment of reproductive organs, length and weight as seen in Fig. 1a (Stehmann 2002).The total lengths and weights (as seen in Table 1 as mean ± SD) of these specimens ranged between 217 cm (i.e. specimen 2) to 260 cm (i.e. specimen 3) and 86 kg (i.e. specimen 3) to 180 kg (specimen 1) respectively. Clasper and testes lengths and weights for each specimen were also tabulated with their respective reproductive indices (i.e. CF and GSI) (as seen in Table 1). The highest CF of 1.11% was recorded for specimen 1 while the lowest CF of 0.41% was recorded for specimen 3. The highest GSI of 0.41% was calculated for specimen 3 while the lowest of 0.18% was calculated for specimen 1.

### Testes light microscopy assessment

**Fig. 3:**
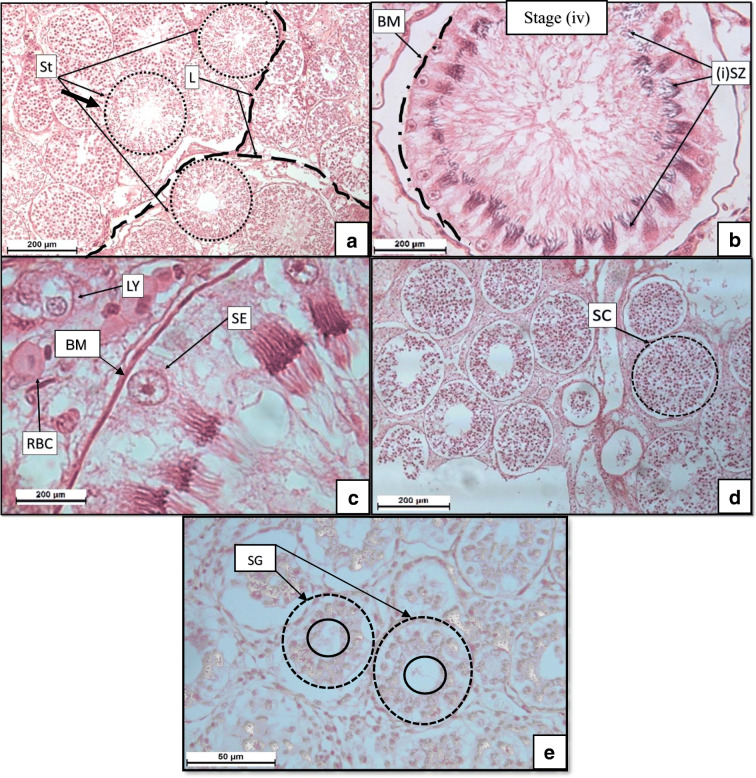
Micrographs showing germ cell structures of the mature *S. lewini*. **a** Testes tissue showing seminiferous tubules (St, represented by dotted circles) located within their respective lobular structure (L, represented by the dashed lines). **b** A closer view of one of the St structures that consists of a basal membrane (BM, represented by a dashed circle enclosing immature spermatozoa (SZ) (which also indicates stage iv). **c** A closer view of the periphery of the basal membrane (BM) of each St structure that contains Sertoli cells (SE) as well as Leydig cells (LY) and red blood cells (RBCs in the medium between St structures). **d** Each St was filled with spermatocytes (SC) identified by the presence of a basophilic sphere-like structures as well as **e** spermatogonia (SG) that have a visible lumen present while forming in the St wherein each rounded structure is formed around the lumen

Light microscopy revealed that the testes tissue of all three examined mature and active male *S. lewini* consisted of lobular structures in various stages of spermatogenesis (i.e. stages i–vii). During the staining process, the term eosinophilic is used to describe the pink stained structures as the dye eosin colours basic structures pink like the cytoplasm. Basophilic is used to describe the purple coloured structures as the dye haematoxylin stains acidic structures purple like the nucleus. Seminiferous tubules were observed as spherical structures (Fig. 3a). The seminiferous tubules contained different stages of basophilic spermatogonia that were found developing towards mature spermatozoa. The eosinophilic basal membrane of the seminiferous tubules and eosinophilic connective tissue were easily distinguished (Fig. 3b).

Sertoli cells were identified as circular structures with darkly stained nuclei (Fig. 3c) along the periphery of the basal membrane. Scattered between the seminiferous tubules were Leydig cells observed as cells with distinctive basophilic nuclei (Fig. 3c). Red blood cells (RBC) were present in the interstitial tissue of the testes and could be identified as eosinophilic cells with distinctive basophilic nuclei (Fig. 3c). Spermatocytes were observed in the seminiferous tubules filled with basophilic cells (Fig. 3d). Spermatogonia were identified at the periphery of the seminiferous tubules easily observed by the clear lumen in the centre of the spermatogonia as the basophilic spheres move outward towards the basal membrane and an empty centre is visible within the seminiferous tubule (Fig. 3e).

### Light microscopy assessment of the development stages of spermatogenesis

**Fig. 4:**
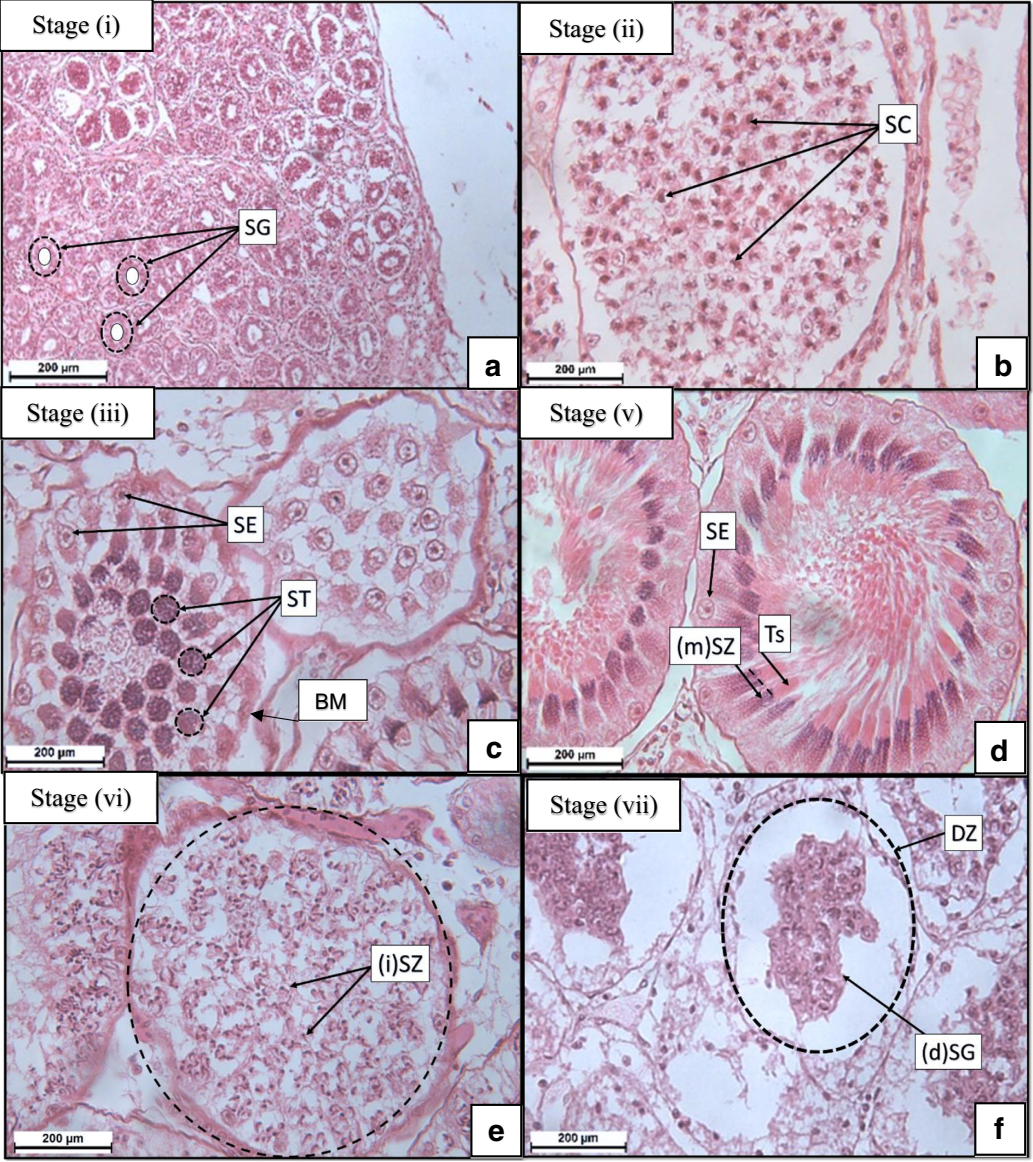
Micrograph**s** of the spermatogenic stages (i–iii, v–vii) in *S. lewini*. **a** Stage (i) shows many spermatogonia (SG, represented by dashed circles). **b** Stage (ii) represents the spermatocytes (SC) that develop from the spermatogonia. **c** Stage (iii) represents the development of spermatids (ST) from the SC as well as Sertoli cells (SE) which support the development. **d** Stage (v) shows mature spermatozoa ((m)(SZ)) identified by typical spiral clusters (spermatozeugmata) at the BM (show the BM) and the clumped eosinophilic tails (Ts). **e** Stage (vi) where immature spermatozoa (i)(SZ) can be seen left behind within the St after mature spermatozoa are spent. **f** Stage (vii) represents the final degeneration of spermatogonia (d)(SG)) seen in the degenerative zone (DZ, indicated by dashed lines)). Stage (iv) can be seen in (**b**) as the (i)(SZ) move to the periphery of the BM before spiralling

This study identified seven distinct stages of spermatogenesis (Fig. 4) similar to the stages in other elasmobranchs males. Stage (i) was identified by the presence of spermatogonia (Fig. 3a). Stage (ii) was notable as spermatocytes which normally develop from the spermatogonia (Fig. 4b). In stage (iii), basophilic spermatids formed/developed in the centre of the seminiferous tubules (Fig. 4c). Stage (iv) showed immature spermatozoa, that stained purple, moving to the periphery of the basal membrane (Fig. 3b). In stage (v), the mature spermatozoa start spiralling on the inner edge of the basal membrane with the Sertoli cells clearly visible and eosinophilic tails of the spermatozoa were present (Fig. 4d). Sperm heads clusters (mentioned in stage (v)), known as spermatozeugmata, were bound in the eosinophilic matrix and forming in a helical shape. Spent mature spermatozoa normally move out of the seminiferous tubules resulting in immature spermatozoa being left behind, as seen in the stage (vi) (Fig. 4e). Finally, stage (vii) shows spermatogonia undergoing degeneration in the degenerative zone.

## Discussion

The macroscopic reproductive anatomy of the mature *S. lewini* male sharks of South Africa is in accordance with existing literature and confirms that the testes of the *S. lewini* are diametrical as seen in other Sphyrnidae and Carcharhinidae (Pratt 1988; Grier 1992; Jensen et al. 2002; Compagno et al. 2005; Conrath 2005; Hamlett 2005; Do Rêgo et al. 2016).

### Biometric data

Biometric data of the three specimens (Table 1) indicated a CF of 1.11% vs. 1.08% for specimen 1 and 2 respectively. A CF > 1% indicates a healthy growth rate (Adams et al. 1993). Specimen 3 with low total mass (86 kg) was the reason this sharks CF (0.49%) was nearly half in weight in comparison with the other specimens regardless of it being the longest specimen (at 260 cm) compared with the rest. It would appear that specimen 3 was undernourished for reasons unbeknown to us as the histology (viewed in this study) appeared healthy similar to the other two specimens, the claspers were stiff upon inspection and the total lengths were in keeping with the literature for mature scalloped hammerhead males (Hazin et al. 2001; Compagno et al. 2005; De Bruyn et al. 2005).

Regardless of the low CF, specimen 3 had the highest GSI (0.41%) followed by specimen 2 (0.35%) and specimen 1 (0.18%). The mean GSI range recorded in late spring (November) for *S. lewini* in KwaZulu-Natal was 0.1–0.35% (De Bruyn et al. 2005) which is similar to this study range (0.18–0.41%). However there has been higher GSI ranges reported in male *S. lewini* by Hazin et al. (2001) where the values ranged between 0.7 and 1.8% during different mating seasons similar to a study by Parsons and Grier (1992) that showed the GSI of *S. tiburo* (bonnethead shark) ranging between 0.2 and 1.6%.

Gonado-somatic index tends to increase from spring to summer and decreases in spring in South Africa (De Bruyn et al. 2005). Late spring (i.e. November) and early summer (i.e. December) are the periods where mature *S. lewini* males are shown to be captured during their inshore movement to breed in late spring/summer (Nov–Feb) (Compagno 1984; Stevens and Lyle 1989; De Bruyn et al. 2005). The turgidity of the testes noticed during the necropsy could have begun to reduce (Parsons and Grier 1992) due to the decrease in GSI as the male *S. lewini* moving into possible post-mating period (De Bruyn et al. 2005).

The GSI of this study’s specimens and those from the same population, as reported by De Bruyn et al. (2005), could have been lower than the values for the same species in Brazil (Hazin et al. 2001) due to the fact that the latter were based on eviscerated weight.

### Histology assessment

The testes tissue from this study corresponded with many previous histological description of diametrical testes (Pratt 1988; Parsons and Grier 1992; Do Rêgo et al. 2016) except for a study done by Bejarano-Álvarez et al. (2010) where spermatocytes and spermatids identified seem indistinguishable from one another. As with other studies, the testes described in this study were surrounded by the epigonal organ and were subdivided into in different stages of development (Pratt 1988; Do Rêgo et al. 2016). According to Grier (1992), the type of spermatogenesis occurring within the elasmobranch testes tissue is polyspermatocystic which was corroborated in this study. Seven stages of spermatogenesis have been described in previous studies (Grier 1992; Do Rêgo et al. 2016) and the same have been found in this study and will be described in the following paragraph.

Spermatogenesis begins in the germinal zone alongside the dorsal-lateral wall of the testes as seen in *S. tiburo* from the Gulf of Mexico (Parsons and Grier 1992). During the process of mitosis (i.e. cell duplication), spermatogonia form alongside the germinal zone and are identified by the clear lumen central area which conforms with what was seen in *S. lewini* (Parsons and Grier 1992) and other species such as the *Prionace glauca* (blue shark)*, Rhizoprionodon lalandii* (Brazilian sharpnose shark) and *Mustelus canis* (smooth hound shark) from the South Atlantic (Do Rêgo et al. 2016). Spermatogonia developed into primary spermatocytes through meiosis (i.e. two genetically unique cells are formed) followed by secondary spermatocytes through a second meiosis which conforms with previous studies (Grier 1992; Parsons and Grier 1992; Girard et al. 2000; Do Rêgo et al. 2016). Spermatocytes were identified in this study during assessment and were observed to develop into spermatids within the seminiferous tubules (Parsons and Grier 1992). This was also observed in *S. tiburo* (Parsons and Grier 1992) and in the diametric testes of deep seawater sharks from the British Isles, *Centroscymnus coelolepis* (Portuguese dogfish) and *Centrophorus squamosus* (leafscale guper shark) (Girard et al. 2000). Spermatids move to the periphery of the seminiferous tubules towards the basal membrane (Grier 1992; Parsons and Grier 1992; Girard et al. 2000; Do Rêgo et al. 2016) to undergo further development to form immature spermatozoa which become embedded in clusters alongside the basal membrane with distinctive Sertoli cells which regulates the spermatozoa (Pratt and Tanaka 1994; Do Rêgo et al. 2016). When the immature spermatozoa began to mature, a structure called the spermatozeugmata is formed due to mature spermatozoa spiralling alongside the basal membrane inside the matrix material. This was confirmed in *S. lewini* caught in the area ranging from the Gulf of Mexico to the Sable Island in Canada (Pratt and Tanaka 1994). Inside the degenerating zone of the testes, the seminiferous tubules were seen as empty where the spermatogonia degenerated in the area and these were also seen in the species from the following studies (Grier 1992; Girard et al. 2000). Spent tubules were found with immature spermatozoa which remained in the tubules.

Leydig cells were identified in the interstitial tissue between tubules in this study. These cells regulate androgen production which play a role in sperm production (Chieffi 1962). The presence of Leydig cells in interstitial tissue in elasmobranchs has been disputed in the past but there has been some evidence of them being found between seminiferous tubules (Chieffi 1962) as well being corroborated in our study. Sertoli cells are found alongside the basal membrane of the seminiferous tubules and play a role in maintaining germ cells becoming more pronounced in the later stages of development as it is associated with reproduction (McClusky 2018) which was also seen in *Carcharhinus limbatus* (blacktip shark) testes (Grier 1992).

Mature spermatozoa were observed in all three specimens of *S. lewini* in both the left and the right testes, this allowed for the confirmation that these males sharks histologically met the criteria of a mature classification as well as being sexually active, further supporting the initial classification which was based on morphometric details. A more detailed description of stages were also provided in this study for the species of *S. lewini* which was similar to previous studies (Pratt 1988; Grier 1992; Parsons and Grier 1992; Pratt and Tanaka 1994; Girard et al. 2000; Bejarano-Álvarez et al. 2010; Do Rêgo et al. 2016).

## Conclusion

This is the first fully detailed description of germ cell development in sexually mature *S lewini* male sharks from South Africa. The seven stages of spermatogenesis, the diametrical and polyspermatocystic testes identified in this study, using adequate histological techniques, conform to the previous literature of elasmobranch males. The histological findings also confirm the sexual staging technique initially taken on this study.

The testes of *S. lewini* consist of seminiferous tubules which form part of a larger lobular structure with germ cells in different stages of development (i–vii), from spermatogonia to mature spermatozoa.
